# Urinary Titin on the First Postoperative Day Predicts Long-Term Skeletal Muscle Loss in Patients with Gastroenterological Cancer

**DOI:** 10.3390/ijms26052026

**Published:** 2025-02-26

**Authors:** Momoko Kyomen, Ayako Tatsumi, Rie Tsutsumi, Yuna Izumi-Mishima, Mizusa Hyodo, Eiji Tanaka, Kohta Iguchi, Kojiro Taura, Hiroaki Terajima, Sachiko Honjo, Akihiro Hamasaki, Kazuhiro Nomura, Hiroshi Sakaue

**Affiliations:** 1 Department of Nutrition and Metabolism, Graduate School of Biomedical Sciences, Tokushima University, Tokushima 770-8503, Japan; 2 Department of Nutrition, Medical Research Institute KITANO HOSPITAL, PIIF Tazuke-Kofukai, Osaka 530-8480, Japan; 3 Department of Anesthesiology, Critical Care Hiroshima University, Hiroshima 734-8551, Japan; 4 Department of Gastroenterological Surgery and Oncology, Medical Research Institute KITANO HOSPITAL, PIIF Tazuke-Kofukai, Osaka 530-8480, Japan; 5 Department of Diabetes and Endocrinology, Medical Research Institute KITANO HOSPITAL, PIIF Tazuke-Kofukai, Osaka 530-8480, Japan; 6 Diabetes Therapeutics and Research Center, University of Tokushima, Tokushima 770-8503, Japan

**Keywords:** urinary titin, skeletal muscle atrophy, gastroenterological cancer

## Abstract

Perioperative malnutrition is common in patients with gastroenterological cancer and contributes to postoperative skeletal muscle atrophy, which adversely affects their prognosis. Early assessment of skeletal muscle atrophy is crucial for improving postoperative outcomes. This study aimed to evaluate the efficacy of urinary titin as a biomarker for skeletal muscle atrophy. A prospective observational study was conducted, and a total of 34 gastroenterological cancer patients were included. Urinary titin levels were measured using ELISA at admission, postoperative days (POD) 1, 7, and 14, and at 6 months after surgery. Surgical procedure, operative time, cancer stage, postoperative complications, hospital stay, and preoperative and postoperative body composition were evaluated, along with nutritional status and grip strength from admission to 6 months after surgery. Changes in urinary titin levels were measured at the same time points as described above. Preoperatively, the mean urinary titin level was 5.03 pmol/mg Cr, slightly higher than in healthy subjects. Urinary titin peaked at 33.71 (24.30–66.58) pmol/mg/dL Cr on POD1 and was associated with serum free branched-chain amino acid concentrations. Urinary titin on POD1 was significantly correlated with a decrease in skeletal muscle mass (rs −0.361, *p* = 0.036) and body cell mass (rs −0.361, *p* = 0.038) at 6 months postoperatively. The grip strength at 6 months postoperatively tended to decrease (rs −0.342, *p* = 0.052). BMI and serum LDH at admission were associated with urinary titin on POD1 but were not correlated with skeletal muscle loss at 6 months, suggesting that urinary titin on POD1 is an independent biomarker of skeletal muscle atrophy. These data indicate that urinary titin on POD1 can predict long-term skeletal muscle atrophy.

## 1. Introduction

Gastroenterological cancer is one of the life-threatening cancers ranking fifth worldwide in terms of both incidence and mortality rates [1]. For patients with gastroenterological cancer, partial or extensive resection and reconstruction of the gastrointestinal tract are often necessary for cancer treatment but can also lead to serious and life-threatening complications such as malnutrition. In fact, the prevalence of malnutrition among gastroenterological surgery patients is 44% [2]. Patients with gastroenterological cancer have a higher rate of malnutrition compared to those with other types of cancer [3]. This may be due to the fact that gastroenterological cancer is primarily located at or often infiltrates into the digestive tract, leading to a reduction in dietary intake, malabsorption, and, ultimately, malnutrition. Since the gastrointestinal tract plays a critical role in the digestion and absorption of nutrients, the loss of its function following resection can result in deficiencies of macronutrients, vitamins, and minerals. These deficiencies can ultimately lead to skeletal muscle atrophy and weight loss [4,5]. Postoperative skeletal muscle atrophy results in decreased physical function, delayed recovery from surgical stress, increased risk of postoperative complications such as infection, and poorer prognosis [6,7].

Postoperative malnutrition, including skeletal muscle atrophy, results in decreased physical function, delayed recovery from surgical stress, increased risk of postoperative complications such as infection, and poorer prognosis [6,7]. Preoperative malnutrition can lead to delayed tissue repair and delayed wound healing at the surgical site, as well as an increased risk of infection due to compromised immune function. It also has a significant impact on patient prognosis, including postoperative complications such as suture failure and pneumothorax, as well as reduced quality of life and activities of daily living due to decreased skeletal muscle mass and skeletal muscle strength. Skeletal muscle atrophy has also been linked to higher rates of cancer recurrence and mortality, and it can increase the incidence of volume-limiting toxicities during postoperative chemotherapy [8,9]. Therefore, early evaluation and prevention of skeletal muscle atrophy are crucial for improving postoperative management, prognosis, and patient quality of life.

Methods for assessing skeletal muscle mass include computed tomography (CT), ultrasound, and bioelectrical impedance analysis (BIA). CT and ultrasound are important diagnostic and treatment tools, but CT is invasive due to radiation exposure, while ultrasound is non-invasive but operator-dependent, which can lead to interrater variability. As a result, BIA has become the most commonly used method for assessing skeletal muscle mass [10]. However, BIA can be prone to error due to the effects of body water, making it challenging to monitor fluctuations during the perioperative period, when intra- and extracellular water content is unstable. Several potential biomarkers of skeletal muscle atrophy have been reported so far. In addition to muscle strength, such as grip strength and walking speed, which are indicators of muscle atrophy, blood vitamin D levels indicate a potential predictor of future muscle atrophy, and creatinine reflects skeletal muscle mass. In addition, it has recently been reported that TDP-43 protein accumulates in intramuscular nerve bundles in skeletal muscle from the early stages of the disease in amyotrophic lateral sclerosis, a disease that causes motor neuron degeneration, muscle atrophy, and muscle weakness. Urinary titin, also examined in this study, is a structural protein of myofibrils [11].

Urinary titin was first identified as a biomarker for diagnosing muscular dystrophy with significant muscle atrophy [11]. Titin is a large sarcomeric protein that functions as a spring, contributing to muscle stretch and elasticity [12,13]. It connects thin filaments containing actin with thick filaments containing myosin [14]. As the largest protein in the human body, titin plays a key role in muscle extension and viscoelasticity [11]. When muscle tissue is damaged, titin is degraded and excreted in the urine. The N-terminal fragment of titin can be measured by Enzyme-Linked Immunosorbent Assay (ELISA) and serves as a biomarker for muscle atrophy [11,14]. We have previously reported that urinary titin can be a biomarker for skeletal muscle atrophy in patients with critical illnesses such as acute stroke, acute myocardial infarction, and diabetes [15,16,17,18]. In intensive care unit (ICU) patients, higher urinary titin levels were associated with a higher incidence of ICU-acquired weakness (ICU-AW) [15]. Additionally, titin is detectable in urine as early as 2 h after the onset of cerebral infarction [16], and elevated titin levels have been observed in patients with acute myocardial infarction [17]. In patients with type 2 diabetes, high titin levels were linked to a higher incidence of disease-related sarcopenia. In surgical patients, invasive cardiovascular procedures have been reported to increase urinary titin concentrations [18].

However, while many studies have suggested a relationship between skeletal muscle atrophy and urinary titin in the context of acute invasive procedures, none have definitively demonstrated a correlation with long-term postoperative outcomes. Therefore, in this study, we focused on patients with gastroenterological cancer, where long-term postoperative weight loss and skeletal muscle atrophy are prognostically significant. The objective of this study was to determine whether urinary titin could serve as a biomarker for long-term postoperative skeletal muscle atrophy and nutritional status by monitoring urinary titin levels and skeletal muscle mass in patients undergoing gastroenterological surgery.

## 2. Results

### 2.1. Patient Characteristics

Forty-one patients were prospectively enrolled in the study. Seven patients were excluded: one patient (2.4%) who was unable to undergo the BIA procedure due to pacemaker placement, one patient (2.4%) who died during the study period, one patient (2.4%) who withdrew consent, and four patients (9.7%) who lacked measurements at 6 months. As a result, 34 patients were included in the final analysis. As shown in Table 1, the mean age was 68.7 ± 8.5 years, with 16 (47.1%) male patients. The mean BMI at admission was 22.3 (20.4–23.8) kg/m^2^. The prevalence of malignancy was 32 patients (94.1%), including 6 (17.6%) with esophageal cancer, 18 (52.9%) with gastric cancer, 7 (20.5%) with pancreatic cancer, and 1 (2.9%) with liver cancer.

### 2.2. Changes in Urinary Titin Levels

**Figure 1:**
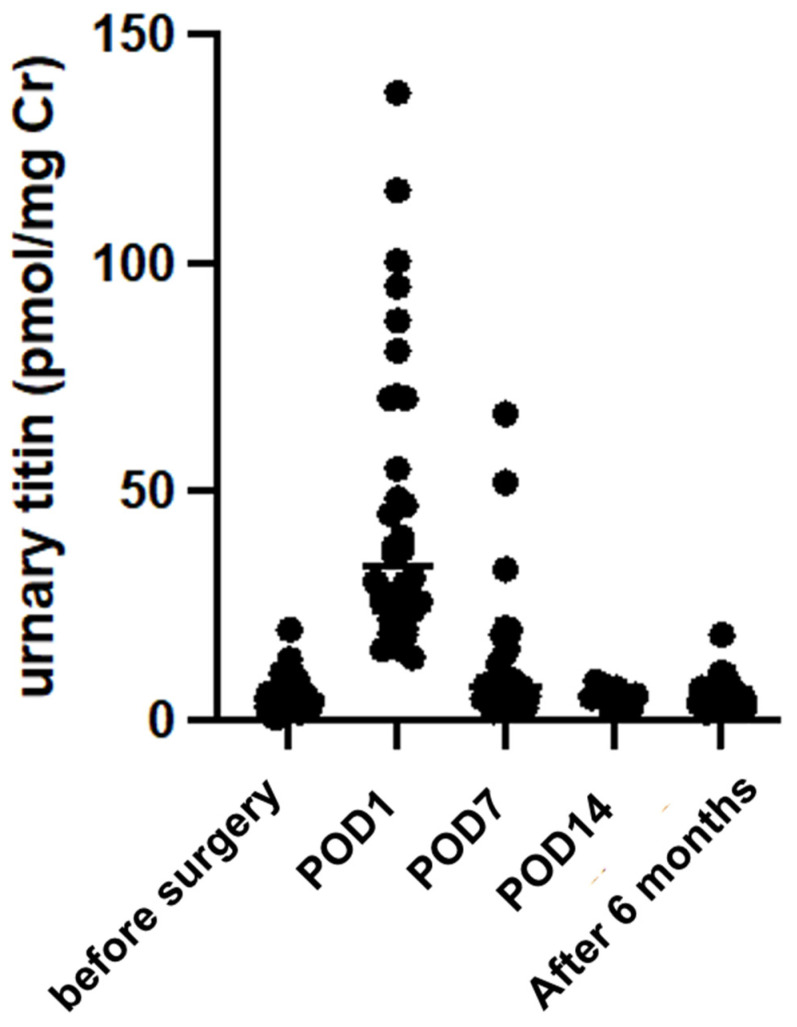
Urinary titin concentration after surgery. Urinary titin was measured on the day of admission, POD1 (day 1), POD7 (day 7), POD14 (day 14), and 6 months postoperatively (after 6 months).

The changes in urinary titin levels from preoperatively to 6 months postoperatively are shown in Figure 1. Preoperatively, urinary titin levels were 5.03 (3.05–8.51) pmol/mg Creatinine (Cr), which was slightly higher than in healthy subjects (1–3 pmol/mg Cr), particularly in patients with esophageal cancer (8.2 ± 3.1 pmol/mg Cr). Urinary titin peaked at 33.71 (24.30–66.58) pmol/mg/dL Cr on postoperative day (POD) 1 and decreased to 7.24 (5.42–12.37) pmol/mg Cr on POD7.

**Figure 2:**
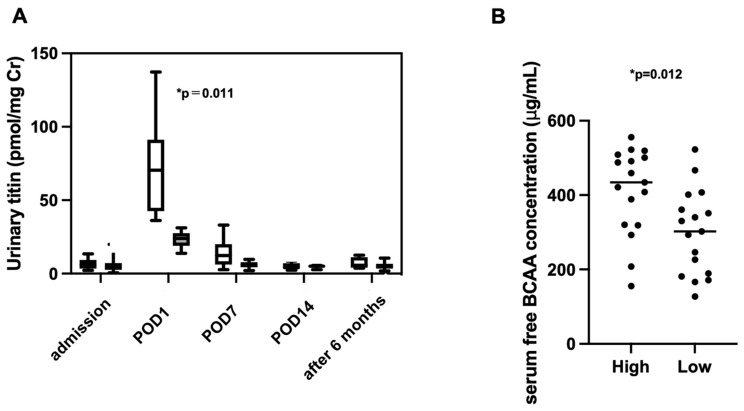
Comparison of change of titin level and free BCAA concentration. (**A**) Comparison of urinary titin level changes after surgery between the high and low urinary titin groups. Open columns represent the high titin group (*n* = 17), and closed columns represent the low titin group (*n* = 17). * *p* < 0.011. (**B**) Serum free BCAA concentrations; high titin group (high) vs. low titin group (low), *n* = 17 each. * *p* = 0.012.

Urinary titin levels on POD1 varied widely among individuals, and factors contributing to this variation were investigated. As shown in Table 2, patients were grouped into two groups based on median urinary titin level (33 pmol/mg Cr) on POD1. The urinary titin concentration in the high titin group was significantly higher only on POD1 (Figure 2A; 70.4 pmol/mg Cr vs. 23.9 pmol/mg Cr, *p* < 0.001) and there was no significant difference in titin level between these two groups after 6 months of surgery (Figure 2A). Additionally, we compared preoperative nutritional status and the extent of surgical invasiveness between the groups. Postoperative nutritional intake was significantly lower on POD7 than preoperatively, but there was no difference between the two groups. BMI in the low titin group was higher than that in the high titin group; however, there were no significant differences in preoperative nutritional assessments (Subjective Global Assessment; SGA, energy intake, skeletal muscle mass, serum protein concentration, and grip strength) between the two groups. Furthermore, there were no differences in surgical time, intraoperative blood loss, malignancy, or tumor stage.

As shown in Figure 2B, serum free branched-chain amino acid (BCAA) concentrations were significantly higher in the high titin group, suggesting a higher degree of hyper-catabolism in this group (620.2 ± 199.0 µg/mL vs. 227.9 ± 104.0 µg/mL, *p* = 0.012). Length of hospital stay tended to be longer in the high titin group (17 days [14–21 days] vs. 20 days [13–25 days] in the high titin group).

### 2.3. Association Between Urinary Titin on POD1 and Nutritional Status at 6 Months Postoperatively

**Figure 3:**
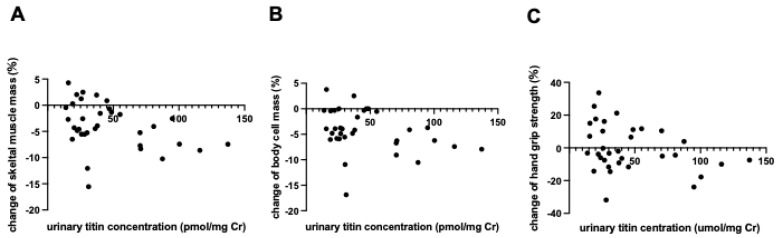
Correlation between urinary titin on the first postoperative day and changes in skeletal muscle mass and function 6 months after surgery. (**A**) Correlation between urinary titin levels on the first postoperative day and the change in skeletal muscle mass after 6 months. (**B**) Correlation between urinary titin levels on the first postoperative day and the change in body cell mass after 6 months. (**C**) Correlation between urinary titin levels on the first postoperative day and the change in hand grip strength after 6 months; *n* = 34, Spearman’s rank correlation was performed.

We next examined the influence of urinary titin on POD1 on nutritional status and other parameters at 6 months after surgery. The relationship between urinary titin on POD1 and body composition, as measured by BIA at 6 months, was assessed. As shown in Figure 3A,B, urinary titin on POD1 was significantly associated with a decrease in skeletal muscle mass at 6 months (rs −0.361, 95% CI [−0.625, −0.034], *p* = 0.036) and body cell mass at 6 months (rs −0.361, 95% CI [−0.614, −0.037], *p* = 0.038). Urinary titin on POD1 tended to correlate with a decrease in grip strength at 6 months after surgery (rs −0.342, 95% CI [−0.613, −0.019], *p* = 0.052) (Figure 3C). Additionally, urinary titin on POD1 was not associated with nutritional indices at 6 months after surgery (serum albumin rs −0.225, 95% CI [−0.559, 0.128], *p* = 0.2, TTR rs −0.154, 95% CI [−0.498, 0.181], *p* = 0.384), or with nutritional intake (energy intake/body weight (kcal); rs 0.11, 95% CI [−0.338, 0.534], *p* = 0.561, protein intake/body weight (g); rs −0.009, 95% CI [−0.416, 0.405], *p* = 0.964).

### 2.4. Factors Associated with Postoperative Elevation of Urinary Titin

To identify factors associated with postoperative elevation of urinary titin, we examined the relationship between blood biochemistry tests, body composition analysis by the BIA method, and preoperative characteristics at admission, including hand grip strength. As shown in Table 3, urinary titin concentration was significantly correlated with serum LDH (*p* < 0.001), serum TTR (*p* = 0.047), and BMI (*p* = 0.028) at admission. Additionally, urinary titin concentration on POD1 was correlated with operative time (*p* = 0.006), intraoperative blood loss (*p* = 0.044), and length of hospital stay (*p* = 0.004). However, urinary titin concentration was not associated with nutritional status, including PNI, preoperative nutrient intake, or underlying diseases.

### 2.5. Factors Associated with Skeletal Muscle Loss 6 Months Postoperatively

To identify risk factors for skeletal muscle loss after 6 months postoperatively, logistic regression analysis was performed, adjusting for covariates such as hospital stay, intraoperative blood loss, and surgical time. As shown in Table 4, urinary titin on POD1 was associated with BMI and serum LDH at admission. When examining the relationship between these factors and skeletal muscle loss after 6 months post-surgery, a stronger association with urinary titin on POD1 was observed (Table 5). On the other hand, no association was found between creatine kinase levels on POD1 and skeletal muscle loss at 6 months (*p* = 0.592).

## 3. Discussion

In this study, we found that high levels of urinary titin on POD1 predicted long-term skeletal muscle atrophy following gastroenterological surgery. In addition to changes in skeletal muscle mass, postoperative urinary titin was also associated with a decrease in body cell mass and hand grip strength 6 months after surgery, suggesting that titin is linked not only to a reduction in muscle volume but also to a decline in muscle function. Urinary titin on POD1 was associated with BMI and serum LDH at admission, but these factors were not associated with skeletal muscle loss at 6 months postoperatively. This suggests that urinary titin on POD1 is the only independent biomarker of skeletal muscle atrophy.

There have been reports on the relationship between skeletal muscle atrophy and urinary titin during the perioperative period [19]. Urinary titin levels increased (43.3 ± 39.5 pmol/mg/dL Cr, *n* = 18) on the day of surgery in cardiac surgery patients, which is comparable to the increase observed in our study. In that report, the increase in urinary titin reflected myocardial damage caused by cardiac surgery. Since muscle damage also occurs during laparotomy in gastroenterological surgery, the influence of laparotomy was also examined in this study. Although the number of cases undergoing laparotomy was small and not fully comparable when the patients were divided into two groups, one with high and one with low POD1, all groups had patients undergoing laparotomy, and there were no significant differences in urine titin levels found between open surgery and laparoscopic surgery. Therefore, although limited, the postoperative increase in urinary titin was not attributed to surgical resection of skeletal muscle but rather to the invasiveness associated with surgical procedures for gastroenterological cancer. Although several reports have shown that urinary titin is an indicator of skeletal muscle atrophy, this is the first report to show that it predicts skeletal muscle atrophy after gastrointestinal surgery, which is one of the most invasive types of cancer resection. This is expected to be a new and important indicator in the preoperative evaluation of gastrointestinal cancer resection.

Various factors may contribute to the increase in urinary titin on POD1 in patients undergoing gastroenterological surgery. Surgery induces catabolism through various proteolytic enzymes, which are activated by inflammatory cytokines, oxidative stress, and increased glucocorticoid levels [4,20]. Muscle protein synthesis is also suppressed under invasive conditions, leading to muscle atrophy due to a catabolic state that exceeds protein anabolism [20,21,22,23]. Furthermore, gastroenterological surgery often involves resection and reconstruction of the digestive tract, which is responsible for nutrient digestion and absorption, and the postoperative diet is often restricted. In the present study, we observed that the high titin group also exhibited increased serum free BCAA levels, which are released from skeletal muscle during muscle degradation. This may suggest that urinary titin levels are influenced by surgical invasiveness, leading to increased catabolism, which could contribute to anorexia in the early postoperative period and reflect changes in postoperative recovery from surgical invasiveness, particularly in skeletal muscle mass.

Recently, we investigated urinary titin as a potential early biomarker for skeletal muscle atrophy across various pathological conditions in mice [24]. Our results demonstrate that urinary titin levels increase in response to muscle damage and catabolism in four different mouse models: acute injury by cardiotoxin, disuse (immobilization), systemic inflammation (lipopolysaccharide-induced sepsis), and metabolic disorder (Streptozotocin-induced diabetes). Importantly, we observed differences in the timing and magnitude of urinary titin elevation across these models, providing insight into the process of muscle catabolism in each condition. The magnitude and timing of titin elevation varied between the chronic and acute phases. In this study, preoperative titin levels in gastroenterological cancer patients were higher than those in healthy individuals, and titin levels were elevated in POD1, suggesting that the degree and cause of catabolism indicated by the high titin levels at these two points are different. Notably, only titin levels on POD1 were significantly correlated with skeletal muscle atrophy 6 months after surgery, indicating that the acute surgical invasiveness influences long-term skeletal muscle atrophy in the postoperative period.

The perioperative period is a critical phase for systemic metabolism. There is broad consensus on the role of nutritional status in gastroenterological cancer staging, surgery, and outcome, and adherence to published guidelines [25,26,27] can facilitate early and standardized management of nutritional status before treatment and ensure that patients receive appropriate nutritional support potential. On the other hand, the optimal approach to nutritional intervention for the catabolism observed in gastrointestinal cancer patients in the perioperative period remains controversial. Although all patients in this study underwent nutritional assessment from admission and received tailored nutritional management, the impact of surgical invasiveness may have been particularly pronounced in the very early postoperative period. Therefore, there may still be opportunities to improve the management of severe catabolism, and timely, targeted nutritional and therapeutic interventions immediately after surgery—guided by appropriate indicators such as elevated urinary titin—could help maintain nutritional status, including the preservation of skeletal muscle mass and muscle strength, over the long term.

Perioperative skeletal muscle loss is associated with poor prognosis, including increased mortality and postoperative complications [6,7,8]. During the perioperative period, edema occurs due to increased vascular permeability, which makes the BIA method unreliable for evaluation and the lower extremity echocardiography method difficult to standardize across institutions from a technical standpoint. CT, while effective, is invasive and unsuitable for assessing skeletal muscle mass in this context. In contrast, urinary titin is a noninvasive marker, as it uses spontaneously excreted urine, with spot measurements also possible [14]. When compared with other tissues, such as the liver and gut, skeletal muscle protein turnover is normally relatively slow, and it only varies slightly during fasting and feeding, with net breakdown and net synthesis. Skeletal muscle atrophy occurs when the rates of protein degradation exceed the rates of protein synthesis, and excess protein degradation is the main cause of muscle dysfunction. In this study, only the urinary titin level was high on POD1, and there was no prolonged release of titin regardless of the magnitude of the surgical invasion. However, considering the characteristics of skeletal muscles that are less susceptible to feeding and fasting, it can be inferred that the acute skeletal muscle degradation observed immediately after surgery has a long-term effect. In addition, these cannot be inferred from standard inflammatory indices such as CRP, and future studies may need to examine the association with more rapid turnover indices, such as IL-6. Therefore, urinary titin holds promise as a novel biomarker for the early detection of skeletal muscle loss.

Creatinine kinase is abundant in abdominal muscles and is elevated following strenuous exercise, muscle injury, or muscle injection. It is also a known biomarker for potential muscle atrophy [23,28]. Therefore, in the present study, we examined whether creatinine kinase, similar to urinary titin, was associated with skeletal muscle atrophy after 6 months. However, creatinine kinase was not a sufficient biomarker. In our previous study, creatinine kinase was found to be unsuitable for identifying ICU-AW or as a biomarker for muscular dystrophy, even in the ICU setting, due to its limited specificity in cases of significant muscle atrophy [15,29]. Muscle atrophy may occur because creatinine kinase, unlike titin, leaks from various tissues and is influenced by factors other than rhabdomyolysis [30]. In addition, titin has only about a 30-h half-life, whereas prealbumin, which is often used for nutritional assessment, has about a 48-h half-life. These findings suggest that urinary titin is a better biomarker for skeletal muscle atrophy than creatinine kinase and prealbumin.

This study has several limitations. First, the sample size is small, and there is a diversity of tumor types included. Because this study required obtaining patient consent at a single facility, a sufficient number of cases could not be collected during the study period. Based on the standard deviation, confidence level, and margin of error of this study, a sample size of approximately 150 cases is required, and further studies with larger samples are needed in the future. Second, we could not consider cancer type, the sites of gastroenterological surgical resection, and the techniques. Even for the same site, reconstruction methods can vary, and the evaluation of the resection area differs between institutions. There was no difference in the distribution of patients by cancer type when divided into the high-titin and low-titin groups, as shown in Table 2. Similarly, there were no differences in Stage or resection site. On the other hand, there was no significant difference in the number of patients who underwent laparotomy, but there were four patients in the high titin group and one patient in the low titin group. Therefore, when increasing the sample size, it is necessary to increase the number of cases of each cancer type and to compare the number of patients who underwent laparotomy with or without laparotomy and the magnitude of surgical invasion. Third, there are differences in factors that affect the postoperative course, such as rehabilitation, activity levels, and the presence or absence of postoperative treatments like chemotherapy. These factors may influence the maintenance or change in postoperative muscle mass, but we were unable to accurately account for them in this study. Despite these limitations, to our knowledge, this is the first study to report that urinary titin predicts long-term postoperative skeletal muscle atrophy in gastroenterological surgical patients. We suggest that urinary titin on POD1 may serve as a biomarker for mid- to long-term risk of skeletal muscle atrophy during the postoperative period of 6 months.

In conclusion, this study demonstrates that high levels of urinary titin on POD1 predict skeletal muscle atrophy at 6 months after surgery and may inform new approaches to improve patient prognosis. Assessment of urinary titin levels in POD1 is predictive of long-term skeletal muscle atrophy; future developments will clarify the effects of interventions such as early postoperative nutrition and rehabilitation interventions for patients with high titin levels and more precise nutrition and rehabilitation follow-up after discharge from the hospital in order to confirm the clinical significance of measuring titin in gastrointestinal cancer patients. This would confirm the clinical significance of titin measurement in patients with gastrointestinal cancer.

## 4. Materials and Methods

### 4.1. Study Design

A prospective observational study was conducted at the Medical Research Institute KITANO HOSPITAL, PIIF Tazuke-Kofukai, from July 2022 to February 2024. The study was approved by the Hospital’s Ethics Committee (approval number: P220200800, approve date 9 February 2022). It adhered to the “Ethical Guidelines for Medical and Health Sciences Research Involving Human Subjects” set by the Ministry of Health, Labor, and Welfare and complied with the provisions of the Declaration of Helsinki. The study was registered as a clinical trial (University Hospital Medical Information Network—Clinical Trial Registration: UMIN-000048021). Written informed consent was obtained from patients or their surrogates at the time of enrollment. All patients were followed up during hospitalization and after 6 months post-surgery. The primary outcome was the diagnostic utility of urinary titin for muscle atrophy after surgery.

### 4.2. Patient Selection Criteria

Patients who underwent upper gastroenterological surgery (subtotal esophagectomy, distal gastrectomy, proximal gastrectomy, or total gastrectomy), hepatectomy, or pancreatic surgery (pancreatic head and tail resection) during the study period were included. The inclusion criteria were patients who were 20 years old and over and provided prior informed consent. Exclusion criteria included the following: (1) patients with muscular dystrophy or other conditions that may cause chronic muscle atrophy or degeneration, and (2) patients undergoing dialysis.

### 4.3. Urinary Titin Measurement

Urinary titin was measured at admission, on the 1st, 7th, and 14th day, and at 6 months after surgery. Titin levels were assessed using an ELISA kit (Human Titin N-Fragment Urine ELISA Kit #29501, Immunobiology Laboratory Co., Ltd., Fujioka, Gunma, Japan), as previously described [15,31]. To account for variations in urine concentration, urinary titin levels were normalized to urinary creatinine concentrations, which were determined using an enzymatic assay (L-type Creatinine M, Fujifilm Wako Pure Chemicals, Osaka, Japan).

### 4.4. Nutritional and Other Assessment

The primary endpoint of the study was the change in skeletal muscle mass at 6 months postoperatively. Secondary endpoints included nutritional status, somatic cell mass, grip strength, perioperative complications, perioperative food intake, intraoperative blood loss, and length of hospital stay at 6 months postoperatively. Additionally, the following data were prospectively collected: disease diagnosis, surgical procedure, operative time, operative blood loss, cancer stage, postoperative complications, hospital stay duration, and preoperative and 6-month postoperative weight and BMI. Body composition was analyzed using the BIA method (Inbody770^®^, Inbody, Tokyo, Japan), and grip strength was measured. Blood and biochemical data, including serum albumin, RTP, RBP, zinc, creatinine kinase, blood urea nitrogen, creatinine, estimated glomerular filtration rate, blood counts, and prognostic indicators (PNIs), were also collected. Nutritional intake was assessed preoperatively, on the seventh postoperative day, and at 6 months postoperatively using daily food records and the Food Frequency Questionnaire (FFQg, Kenpakusha, Tokyo, Japan) method.

### 4.5. Measurement of Metabolites

Serum samples were mixed with 1 mL of methanol for LC/MS analysis, containing 1 μL of internal standard solution 1 (Agilent Technologies, Tokyo, Japan), 1 mL of chloroform for LC/MS analysis, and 400 μL of distilled water. The mixture was stirred for approximately 30 s and then centrifuged at 2150× *g* for 5 min at 4 °C. The resulting aqueous layer was washed and transferred onto an ultrafiltration filter (Ultrafree MC-PLHCC 250/pk for Metabolome Analysis; Agilent Technologies). The aqueous layer was then aliquoted onto the ultrafiltration filter after washing and centrifuged at 9100× *g* for 4 h at 4 °C. Following centrifugation, the filter cup was removed. The collection tubes were dried in a centrifugal dryer with the lid open for 2 h and stored at −80 °C until analysis. CE-TOFMS (Agilent Technologies) was used for analysis. Immediately prior to analysis, three sample tubes were reconstituted in 50 μL of distilled water with internal standard solution 3 (Agilent Technologies).

### 4.6. Statistical Analysis

All statistical analyses were performed with EZR V 1.68 (Saitama Medical Center, Jichi Medical University, Saitama, Japan), which is a graphical user interface for R (≥4.2.0) (The R Foundation for Statistical Computing, Vienna, Austria). More precisely, it is a modified version of R commander designed to add statistical functions frequently used in biostatistics. Continuous and normally distributed data were presented as mean values with standard deviation (SD), while non-normally distributed data were presented as median values with interquartile ranges (IQR) and categorical data as numbers (%). Non-normally distributed data were compared with the Mann–Whitney U test, and Fisher’s exact test was used to evaluate differences in surgical procedures within each group. Univariate analysis was performed to assess the association of urinary titin on POD1 with admission and to identify risk factors for skeletal muscle atrophy at 6 months postoperatively using Spearman’s correlation coefficient and logistic regression analysis. The association between urinary titin and external variables was evaluated by multiple linear regression. Multiple regression analyses were log-transformed, and data analyses were conducted using EZR. All statistical tests were two-tailed, and a *p*-value < 0.05 was considered statistically significant.

## Figures and Tables

**Table 1:** Patient characteristics.

	*n* = 34
Age (years) (mean ± SD)	68.7 ± 8.5
Male, *n* (%)	16 (47.1)
BMI (kg/m^2^)	22.3 (20.4–23.8)
Length of hospital stay (days)	17.0 (13–23)
Malignancy rate, *n* (%)	32 (94.2)
Esophageal cancer, *n* (%)	6 (17.7)
Gastric cancer, *n* (%)	18 (53.0)
Pancreatic cancer, *n* (%)	7 (20.6)
Liver cancer, *n* (%)	1 (2.9)
Gastrointestinal stromal tumor of the stomach, *n* (%)	1 (2.9)
Mucinous cystic neoplasm of pancreas, *n* (%)	1 (2.9)
Stage	
Stage 0, *n* (%)	3 (9.7)
Stage I, *n* (%)	17 (54.8)
Stage II, *n* (%)	9 (29.0)
Stage III, *n* (%)	2 (6.5)
Co-morbidity	
Hypertension, *n* (%)	15 (44.1)
Diabetes, *n* (%)	8 (23.5)
Dyslipidemia, *n* (%)	8 (23.5)
Liver disease, *n* (%)	8 (23.5)
Hyperuricemia, *n* (%)	5 (14.7)
Renal failure, *n* (%)	0 (0)
Resection site	
Esophagus, *n* (%)	6 (17.7)
Stomach, *n* (%)	DG; 15 (44.2), PG; 3 (8.8), TG; 1 (2.9)
Pancreas, *n* (%)	DP; 3 (8.8), PD; 5 (14.7)
Liver, *n* (%)	1 (2.9)
Surgery	
Laparotomy, *n* (%)	5 (14.7)
Intraoperative hemorrhage (mL)	25.0 (7–125)
Surgical time (h)	6.8 (4.6–9.5)

BMI—body mass index; DG—distal gastrectomy; DP—distal pancreatectomy; PD—pancreaticoduodenectomy; PG—proximal gastrectomy; TG—total gastrectomy.

**Table 2:** Comparison of urinary titin change and patients’ characteristics in the high and low titin groups of POD1.

	POD1 High Titin Group (*n* = 17)	POD1 Low Titin Group (*n* = 17)	*p*-Value
Age (years), (mean ± SD)	68.8 (±10.3)	68.6 (±6.9)	0.973
Male, *n* (%)	6 (35.3)	10 (58.8)	0.303
BMI (kg/m^2^)	20.3 (18.2–22.5)	22.8 (22.0–24.0)	**0.009**
Length of stay, (days)	20 (13–25)	17 (14–21)	0.522
Preoperative assessment			
Serum albumin (g/dL)	3.9 (3.7–4.1)	3.8 (3.6–4.4)	0.616
Serum transthyretin (mg/dL)	23.8 (21.0–26.3)	25.5 (21.1–28.3)	0.614
Serum zinc (μg/dL)	68 (66.8–79.5)	73.0 (68.0–77.0)	0.705
Preoperative body composition			
Skeletal muscle mass (kg)	19.9 (17.9–25.7)	25.4 (20.5–28.3)	0.060
Body cell mass (kg)	24 (21.9–30.4)	30.1 (24.7–33.3)	0.058
Grip strength (kg)	19.6 (17.3–24.0)	24.1 (20.6–29.3)	0.081
Preoperative nutritional screening			
SGA A	16	16	1.000
SGA B	0	1	1.000
SGA C	1	0	1.000
Nutritional intake			
Preoperative			
Energy intake (kg/kcal)	31.4 (26.3–37.8)	29.7 (26.0–31.9)	0.339
Protein intake (g/kg)	1.2 (1.0–1.5)	1.2 (1.0–1.4)	0.563
POD 7			
Energy intake (kg/kcal)	20.8 (16.2–28.6)	21.0 (14.7–24.6)	0.540
Protein intake (g/kg)	1.1 (0.7–1.2)	0.9 (0.7–1.0)	0.197
Stage			0.622
Stage 0, *n* (%)	2 (12.5)	1 (6.7)	
Stage I, *n* (%)	9 (56.3)	8 (53.3)	
Stage II, *n* (%)	5 (31.3)	4 (26.7)	
Stage III, *n* (%)	0 (0)	2 (13.3)	
Malignant tumor, *n* (%)	16 (94.1)	16 (94.1)	0.800
Esophageal cancer, *n* (%)	4 (23.5)	2 (11.8)	
Gastric cancer, *n* (%)	8 (47.1)	10 (58.8)	
Pancreatic cancer, *n* (%)	4 (23.5)	3 (17.6)	
Liver cancer, *n* (%)	0 (0)	1 (5.9)	
Gastrointestinal stromal tumor of the stomach, *n* (%)	1 (5.9)	0 (0)	
Mucinous cystic neoplasm, *n* (%)	0 (0)	1 (5.9)	
Resection site			0.264
Esophagus, *n* (%)	4 (23.5)	2 (11.8)	
Stomach, *n* (%)	DG; 8 (47.1), PG; 1 (5.9), TG; 0 (0)	DG; 7 (41.1), PG; 2 (11.8), TG; 1(5.9)	
Pancreas, *n* (%)	DP; 0 (0), PD; 4 (23.5)	DP; 3 (17.6), PD; 1 (5.9)	
Liver, *n* (%)	0 (0)	1 (5.9)	
Surgery			
Laparotomy, *n* (%)	4 (23.5)	1 (5.9)	1.000
Intraoperative hemorrhage (mL)	25.0 (15–125)	25.0 (5.8–99.5)	0.829
Surgical time (h)	8.0 (4.0–10.2)	6.2 (5.1–8.7)	0.760

DG—distal gastrectomy; DP—distal pancreatectomy; PD—Pancreaticoduodenectomy; PG—proximal gastrectomy; POD—postoperative day; TG—total gastrectomy.

**Table 3:** Factors associated with urinary titin on the first postoperative day: Simple linear regression analysis.

	Univariate Analysis				
Variables	Partial Regression Coefficient	95% CI	Standard Error	t-Statistic	*p*-Value
Serum albumin (g/dL)	−3.020	(−28.665–22.626)	12.590	−0.240	0.812
Serum c-reactive protein (mg/dL)	−35.461	(−110.463–39.542)	36.821	−0.963	0.343
Serum creatine kinase (IU/L)	0.064	(−0.243–0.3704)	0.150	0.425	0.673
Serum lactate dehydrogenase (U/L)	0.574	(0.267–0.882)	0.151	3.801	**<0.001**
Serum cholinesterase (IU/L)	0.011	(−0.142–0.164)	0.075	0.150	0.882
Hemoglobin (g/dL)	−4.005	(−9.587–1.576)	2.740	−1.462	0.154
Serum transthyretin (mg/dL)	1.424	(0.023–2.826)	0.687	2.073	**0.047**
Serum transferrin (mg/dL)	0.124	(−0.125–0.373)	0.122	1.013	0.319
Serum retinol-binding protein (mg/dL)	9.251	(−0.655–19.158)	4.857	1.905	0.066
Serum zinc (μg/dL)	0.378	(−0.516–1.272)	0.438	0.863	0.395
Urinary titin at the time of admission (pmol/mg Cr)	0.757	(−2.053–3.568)	1.380	0.549	0.587
BMI (kg/m^2^)	−4.470	(−8.428–0.512)	1.943	−2.301	**0.028**
Grip strength (kg)	−0.986	(−2.630–0.657)	0.806	−1.224	0.230
Skeletal muscle mass (kg)	−1.826	(−4.121–0.469)	1.127	−1.621	0.115
Body cell mass (kg)	−1.673	(−3.764–0.417)	1.026	−1.631	0.113
Body fat percentage (%)	0.127	(−1.495–1.749)	0.797	0.159	0.874
ECW/TBW	405.922	(−547.519–1359.37)	468.077	0.867	0.392
Phase Angle	−11.745	(−25.8019–2.3113)	6.901	−1.702	0.098
Length of stay (days)	1.282	(0.447–2.118)	0.410	3.125	**0.004**
Age (years)	−1.139	(−2.418–0.139)	0.628	−1.816	0.079
Complications	21.401	(−6.956–49.758)	13.921	1.537	0.134
Surgical time (h)	4.521	(1.417–7.626)	1.524	2.967	**0.006**
Intraoperative blood loss (mL)	0.052	(0.001–0.103)	0.025	2.097	**0.044**

BMI—body mass index; ECW/TBW—extracellular water/total body water.

**Table 4:** Factors associated with the increase in urinary titin on POD1.

	Model 1		Model 2		Model 3	
	OR (95% CI)	*p*-Value	OR (95% CI)	*p*-Value	OR (95% CI)	*p*-Value
Length of stay (days)	1.00 (0.88–1.13)	0.971	1.05 (0.89–1.22)	0.563	1.05 (0.96–1.15)	0.263
Intraoperative blood loss (mL)	1.00 (0.99–1.00)	0.566	1.00 (0.99–1.00)	0.621	0.10 (0.99–1.00)	0.680
Surgical time (h)	1.41 (0.85–2.35)	0.184	1.10 (0.71–1.70)	0.671	1.06 (0.73–1.53)	0.761
BMI at admission (kg/m^2^)	0.53 (0.31–0.91)	**0.022**				
Serum lactate dehydrogenase at admission (U/L)			1.05 (1.01–1.09)	**0.015**		
Serum transthyretin at admission (mg/dL)					0.10 (0.89–1.12)	0.942

**Table 5:** Factors associated with skeletal muscle loss at 6 months postoperatively.

	OR (95% CI)	*p*-Value
BMI at admission (kg/m^2^)	1.07 (0.76–1.5)	0.711
Serum lactate dehydrogenase at admission (U/L)	0.96 (0.92–1.0)	0.054
Urinary titin on postoperative day 1 (pmol/mg Cr)	1.05 (1.01–1.1)	**0.026**

BMI—body mass index.

## Data Availability

Data are available upon reasonable request for academic non-commercial research purposes.
